# Prescription rate of medications potentially contributing to lower urinary tract symptoms and detection of adverse reactions by prescription sequence symmetry analysis

**DOI:** 10.1186/s40780-014-0004-1

**Published:** 2015-02-15

**Authors:** Masako Hashimoto, Kanako Hashimoto, Fumihiko Ando, Yoshiaki Kimura, Keisuke Nagase, Kunizo Arai

**Affiliations:** Faculty of Pharmacy, Institute of Medical, Pharmaceutical and Health Sciences, Kanazawa University, Kakuma-machi, Kanazawa, 920-1192 Japan; Temari Pharmacy, 2-50 Kobu-machi, Kanazawa, 920-0362 Japan; Department of Medical Informatics, Kyoto University Hospital, 54 Kawaharacho, Syogoin, Sakyu-ku Kyoto, 606-8507 Japan; Department of Medical Informatic, Kanazawa University Hospital, 2-251 Takara-machi, Kanazawa, 920-8641 Japan; Suisen Pharmacy, Fukui Pharmaceutical Association, 906 Matsuokagokuryo Yoshida-gun Eiheijicho-cho, Fukui, 910-1193 Japan

**Keywords:** Lower urinary tract symptoms, Voiding symptoms, Storage symptoms, Prescription sequence symmetry analysis, Adverse reactions

## Abstract

**Background:**

The lower urinary tract symptoms (LUTS) increases with age and can have a significant effect on the quality of life of the patients. Elderly patients, who are often characterized by a decline in physiological functional and polypharmacy, are susceptible to adverse drug reactions to pharmacotherapy. LUTS can also be a side effect of medication. The purpose of this study was to investigate the possible association between the initiation of LUTS-causing drug therapy and the onset of LUTS.

**Methods:**

Drug dispensing data at the individual level were retrieved from the CISA (Platform for Clinical Information Statistical Analysis: http://www.cisa.jp) database. A retrospective study was conducted by reviewing patients with LUTS who were dispensed drugs that increased the risk of LUTS between April 2011 and March 2012. Prescription sequence symmetry analysis (PSSA) was employed to investigate the associations between the dispensing of medicines of LUTS and that of LUTS-causing drugs.

**Results:**

LUTS-causing drugs were frequently dispensed to patients with LUTS. The use of medications potentially contributing to LUTS was associated with polypharmacy [number of prescription drugs:12.13 ± 6.78 (user) vs. 5.67 ± 5.24 (nonuser)] but not patient age [ age: (71.38 ± 13.28 (user) vs. 70.45 ± 14.80 (nonuser)]. Significant adverse drug events were observed the use of donepezil, cyclophosphamide, antiparkinson drugs, antidepressant, diazepam, antipsychotic drugs for peptic ulcer, tiotropium bromide, and opioids.

**Conclusions:**

The use of prescription LUTS-causing drugs was correlated with polypharmacy. The adverse drug events associated with LUTS-causing drugs were highly prevalent in elderly patients. To prevent of adverse drug events in patients with LUTS, pharmacists and physicians should regularly review medication lists and reduce the prescribed medicines.

**Electronic supplementary material:**

The online version of this article (doi:10.1186/s40780-014-0004-1) contains supplementary material, which is available to authorized users.

## Background

The prevalence of lower urinary tract symptoms (LUTS) include voiding symptoms and storage symptoms. Voiding symptoms and storage symptoms are conditions in which the bladder is unable to store urine properly (storage symptoms) or empty properly (voiding symptoms) [1]. Storage symptoms can show as stress incontinence, urge incontinence, mixed storage symptoms, or overflow incontinence. Voiding symptoms presents as a wide range of symptoms that can include difficulty in bladder emptying, urinary hesitancy, slow or weak urine stream urinary retention and dribbling of urine. In men, these symptoms can also be because of an enlarged prostate, a condition known as benign prostatic hypertrophy (BPH).

The LUTS increases with age and can have a significant effect on the quality of life [2]. Older individuals frequently have comorbid disorders and take medications that can impair continence via direct effects upon lower urinary tract function, alteration in volume status and urine excretion, cognitive impairment, and impairment of the functional ability to use a toilet. The hospitalized elderly with incontinence are more likely to have impaired mobility, dementia, and delirium and receive psychoactive medications than their continent patients [3].

Elderly patients, who are often characterized by a decline in physiological function and polypharmacy, are susceptible to adverse drug reactions to pharmacotherapy [4,5]. Poor compliance is most likely to occur among nonhospitalized patients who receive long-term prescriptions of preventive medications. In addition, 40%–50% of elderly patients have been unable to take medication as prescribed by their physicians [6,7].

LUTS can also be a side effect of medication [8,9]. Recently, significant associations between the initiation of calcium channel blockers, angiotensin-converting enzyme inhibitors, angiotensin II receptor blockers, hormone replacement therapies, and hypnotic-sedatives and the subsequent initiation of oxybutynin, which is used to manage urgency incontinence, were reported [10]. Moreover, drugs that induce incontinence include alpha 1-adrenoceptor antagonists, antipsychotics, benzodiazepines, antidepressants, and hormone replacement therapies in postmenopausal women [11-13].

Several studies illustrated that individuals with urinary incontinence are more likely to be using certain medicines, including antihistamines, beta agonists, angiotensinII receptor blockers, anticonvulsants, and benzodiazepines [14]. But a causal link between the use of these medicines and urinary incontinence has not been established.

We examined the prescription rate of drugs that can cause LUTS to patients who ultimately develop LUTS. In addition, we performed prescription sequence symmetry analysis (PSSA) to investigate the risk of LUTS caused by drug side effects.

## Methods

### Study design and data source

Drug dispensing data at the individual level were retrieved from the CISA (Platform for Clinical Information Statistical Analysis: http://www.cisa.jp) database, which contains substantial clinical information obtained in Japan. Currently, data from CISA are provided in a fully anonymized form to 14 national university hospitals. In the CISA database, each prescription record contains basic patient characteristics (anonymous identifier, gender, and date of birth) and information on the drug name, anatomical therapeutic chemical code, dosage, and dispensing date.

This study identified patients with LUTS who were coded according to the 10th revision of the International Statistical Classification of Diseases and Related Health Problems. Medicines known to cause LUTS were identified from drug information resources. The Medicines Handbook [15] was searched to identify medicines with the adverse drug reaction “LUTS.” Finally, published articles were identified and reviewed to identify any additional medicines associated with LUTS. The list of medicines associated with LUTS that were included in the study is presented in Table 1.

**Table 1:** **Number of patients with LUTS prescribed medicines that can cause LUTS**

**(a) Medicines that caused the storage symptoms**			
**Medication class**	**Medicine**	**Prevalence n (%)**	
**Anti-dementia drugs**	Donepezil	272	14.1
**Intestinal Lavage Solution**	Intestinal Lavage Solution	44	2.3
**Decongestants and antiallergics**	Tranilast	18	1.7
	Ketotifen	15	
	other	※	
**Antineoplastic agents**	Cyclophosphamide	17	1.6
**(b) Medicines that caused the voiding symptoms**			
**Medication class**	**Medicine**	**Prevalence n(%)**	
**Anti-parkinson drugs**	Trihexyphenidyl	32	13.7
	Biperiden	56	
	Amantadine	93	
	Levodopa/Benserazide	72	
	other	※	
**Antidepressants**	Mianserin	58	13.8
	Paroxetine e	119	
	Fluvoxamine	61	
	Milnacipran	29	
**Anxiolytics**	Diazepam	95	4.9
	other	※	
**Antipsychotics**	Chlorpromazine	30	13.3
	Risperidone	108	
	Levomepromazine	36	
	Sulpiride	68	
	other	※	
**Drugs for peptic ulcer and gastro-oesophageal reflux disease (GORD)**	Cimetidine	34	5.6
	Scopolamine butylbromide	67	
	other	※	
**Other drugs for obstructive airway diseases, inhalants**	Tiotropium bromide	129	6.7
	other	※	
**Antihistamines for systemic use**	Diphenhydramine	17	3.3
	Chlorpheniramine	40	
	other	※	
**Antiarrhythmics, class I and III**	Cibenzoline	27	2.0
	other	※	
**Cardiac stimulants excl. cardiac glycosides**	Amezinium metilsulfate	20	4.8
	Droxidopa	48	
	Midodrine	24	
	other	※	
**Opioids**	Morphine	19	12.4
	Codeine phosphate	39	
	Oxycodone	172	
	other	※	

※The number of patients prescribed the drug was less than 10.

Medications for LUTS were classified three groups, drugs for the management of storage symptoms, drugs for the management of voiding symptoms, and drugs for the management of mixed urinary dysfunction, as shown in Additional file 1.

A retrospective study was conducted by reviewing patients with LUTS who were dispensed drugs associated with LUTS between April 2011 and March 2012.

The data were stratified to determine the number of drugs and/or the potential LUTS risk of such drugs used by patients with LUTS. To determine whether associations could be detected between the number of medications and age against the use of LUTS-causing medication, Student’s *t*-test was performed to compare the use and nonuse of targeted medications potentially causing or exacerbating LUTS.

### PSSA

PSSA, as developed by Hallas, is an effective surveillance tool for drug-associated adverse reactions [16,17]. PSSA has also been employed in previous studies investigating associations between the uses of certain targeted drugs. The validity of PSSA was confirmed by previous research [18-22].

In PSSA, the date of the first prescription of an LUTS-causing drug (index drug) and that of the first medication used to manage LUTS (marker drug) are determined for each patients. A 6-month “waiting time” was imposed as a baseline period to ensure that the marker drug was indeed newly prescribed. The ratio of the number of patients with initiated LUTS-causing drug therapy before the initiation of LUTS treatment was compared with the number of patients who initiated LUTS-causing drug therapy after the start of LUTS treatment.

### Statistical analysis for PSSA

The ratio of patients who initiated marker drug treatment after the initiation of index drug treatment (index → marker) to those who initiated marker drug treatment before the initiation of index drug treatment (marker → index) was defined as the crude sequence ratio (SR). PSSA could be sensitive to prescribing trends, such as rapid increases in marker drug use, over time. To adjust for such temporal trends, a null-effect SR (*SRnull*) was calculated. *SRnull* is the expected SR in the absence of a causal association, given the incident medicine use and events in the background population. *SRnull* is the expected SR of an incidence trend when there is no causal relationship between the index and marker drugs, providing a background rate for the chronological sequence of two drugs. In this study, we computed the probability of an index drug to marker drug sequence for each user of an LUTS drug at the first prescription. The overall probability of a medicine for treating LUTS, *Pa*, was generated by weighting the number of incident users on each prescribing date of drugs for LUTS and averaging the value over all days. *SRnull* was then computed as *Pa*/(1 – *Pa*). An adjusted SR was obtained by dividing the crude SR by *SRnull*, and 95% confidence intervals (CIs) were determined with a normal approximation to the binomial distribution. All analyses were performed using Ekuseru-Toukei 2012 (Social Survey Research Information Co., Ltd. Tokyo, Japan).

### Ethics statement

The study was approved by the Institutional Review Board of CISA (receipt number: HA1405004).

## Results

### Characteristics of the participants (age, sex, number of drug prescriptions)

Table 2 shows the characteristics of the participants and distribution of the types of LUTS. The mean age of the patients was 70.52 ± 14.60 years, and patients consumed an average of 6.16 ± 5.38 different medications. The proportion of male patients with voiding symptoms was large (91.5%). In men, these symptoms can also be because of BPH.

**Table 2:** **Patient characteristics and frequency of use of medications potentially contributing to urinary symptoms**

	**All**	**Storage symptoms**	**Voiding symptoms**	**Mixed urinary symptoms**
**Number of patients (%)**	17,824 (100%)	5,127 (28.8%)	10,967 (61.5%)	1730 (9.7%)
**Male (%)**	13,777 (77.2%)	2,159 (42.1%)	10,031 (91.5%)	1,587 (91.7%)
**Age (Mean ± SD)**	70.52 ± 14.60	64.90 ± 20.00	72.56 ± 10.98	74.22 ± 10.60
**Number of prescription drugs (Mean ± SD)**	6.16 ± 5.38	6.04 ± 5.58	6.01 ± 5.26	7.50 ± 5.27

### Percentage of prescription agents that were responsible for urinary disturbance and the efficacy classification

The proportion of patients who were using medications potentially contributing to urinary symptoms was 7.7%, with a mean of one incriminating drug per patient (Table 3). The top five medication classes were donepezil (14.1% in user of LUTS-causing drugs), antiparkinson drugs (13.7%), antidepressants (13.8%), antipsychotics (13.3%), and opiates/narcotics (12.4%) (Table 1).

**Table 3:** **Relationship between age and number of medications used for patients prescribed medications potentially contributing to urinary symptoms**

	**All**			**Storage symptoms**			**Voiding symptoms**			**Mixed urinary symptoms**		
	**user**	**nonuser**	**P value**	**user**	**nonuser**	**P value**	**user**	**nonuser**	**P value**	**user**	**nonuser**	**P value**
**Number of patients (%)**	1,365 (7.7%)	16,459	-	234 (4.6%)	4,893	-	907 (8.3%)	10,060	-	224 (12.9%)	1,506	-
**Age (Mean ± SD)**	71.38 ± 13.28	70.45 ± 14.80	0.0244	77.35 ± 10.88	64.30 ± 20.34	<0.0001	69.79 ± 13.58	72.81 ± 10.71	<0.0001	71.63 ± 12.56	74.60 ± 10.27	0.0002
**Number of prescription drugs (Mean ± SD)**	12.13 ± 6.78	5.67 ± 5.24	<0.0001	9.95 ± 6.55	5.86 ± 5.53	<0.0001	12.47 ± 6.82	5.43 ± 5.10	<0.0001	13.05 ± 6.43	6.67 ± 5.08	<0.0001

### The relationships of patient age and polypharmacy with the use of medications potentially contributing to urinary symptoms

The use of medications potentially contributing to LUTS was associated with polypharmacy (12.13 ± 6.78 vs. 5.67 ± 5.24, P value <0.0001) but not patient age (71.38 ± 13.28 vs. 70.45 ± 14.8, P value =0.0244). Among patients with voiding symptoms, those who used LUTS-causing drugs were younger than nonusers (69.79 ± 13.58 vs. 72.81 ± 10.71, P value <0.0001). An opposing trend was observed among patients with storage symptoms (77.35 ± 10.88 vs. 64.30 ± 20.34, P value <0.0001) (Table 3).

### Likelihood of incident medicine after initiation of a medicine that may be associated with LUTS

PSSA identified significant associations between the initiation of opioids [oxycodone (adjusted SR: 1.20; 95% CI: 1.03–1.41), morphine (adjusted SR: 1.29; 95% CI: 1.14–1.45)], donepezil (adjusted SR: 1.98; 95% CI: 1.57–2.50), intestinal lavage solution (adjusted SR: 1.86; 95% CI: 1.65–2.10), cyclophosphamide (adjusted SR: 1.52; 95% CI: 1.14–2.04), levodopa/benserazide (adjusted SR: 1.82; 95% CI: 1.18–2.81), selective serotonin reuptake inhibitor (SSRI) [paroxetine (adjusted SR: 1.77; 95% CI: 1.33–2.36)], serotonin and norepinephrine reuptake inhibitor (SNRI) [milnacipran (adjusted SR: 2.10; 95% CI: 1.28–3.45)], diazepam (adjusted SR: 1.73; 95% CI: 1.46–2.06), serotonin dopamine antagonist [risperidone (adjusted SR: 1.55; 95% CI: 1.34–1.79)], levomepromazine (adjusted SR: 2.20; 95% CI: 1.34–1.79), sulpiride (adjusted SR: 1.32; 95% CI: 1.01–1.72), histamine H2-receptor antagonists [cimetidine (adjusted SR: 1.99; 95% CI: 1.24–3.20)], scopolamine butylbromide (adjusted SR: 1.72; 95% CI 1.55–1.92), anticholinesterases (tiotropium bromide (adjusted SR: 1.75; 95% CI: 1.42–2.16), cibenzoline (adjusted SR: 2.97; 95% CI: 1.92–4.59), and amezinium metilsufate (adjusted SR: 1.89; 95% CI: 1.10–3.26) and the subsequent initiation of medication for managing LUTS (Table 4). When the time window among the events was restricted to 3 months, the results were largely consistent with the primary analyses.

**Table 4:** **Likelihood of incident medicine after initiation of a medicine that may be associated with LUTS (April 2006–January 2014)**

	**Medicine**	**Users of medicine**	**In the 12 months sequence**			**In the 3 months sequence**		
			**Index → Marker/Marker → Index (n)**	**Adjusted SR**	**95% CI**	**Index → Marker/Marker → Index (n)**	**Adjusted SR**	**95% CI**
**Anti-dementia drugs**	Donepezil	7,825	210/109	1.98*	1.57–2.50	175/68	1.32	1.00–3.50
**Intestinal Lavage Solution**	Intestinal Lavage Solution	54,014	722/404	1.86*	1.65–2.10	528/228	1.17	1.06–2.82
**Decongestants and antiallergics**	Tranilast	7,161	0/3	NA	NA		NA	NA
	Ketotifen	4,644	0/0	NA	NA		NA	NA
**Antineoplastic agents**	Cyclophosphamide	7,787	111/77	1.52*	1.14–2.04	64/62	1.42	0.768–1.55
**Antiparkinson**	Trihexyphenidyl	1,844	24/21	1.14	0.63–2.04	21/13	2	0.804–3.21
	Biperiden	3,177	59/41	1.39	0.93–2.07	62/29	1.55	1.33–3.12
	Amantadine	2,155	99/65	1.53*	1.12–2.09	86/47	1.42	1.29–2.62
	Levodopa/Benserazide	1,652	58/32	1.82*	1.18–2.81	49/15	1.78	1.84–5.86
**Antidepressants**	Mianserin	1,999	73/79	0.94	0.68–1.29	71/65	1.4	0.794–1.56
	Paroxetine	9,021	139/70	1.77*	1.33–2.36	120/57	1.37	1.37–2.57
	Fluvoxamine	5,178	65/39	1.48	0.99–2.21	62/28	1.56	1.26–3.08
	Milnacipran	2,578	52/22	2.10*	1.28–3.45	49/13	1.84	1.82–6.18
**Anxiolytic**	Diazepam	57,776	658/450	1.44*	1.28–1.63	602/336	1.14	1.55–2.02
**Antipsychotic**	Chlorpromazine	4,448	121/117	1.09	0.85–1.41	114/91	1.32	1.00–1.74
	Risperidone	14,330	466/310	1.55*	1.34–1.79	491/276	1.16	1.58–2.12
	Levomepromazine	2,391	59/26	2.20*	1.34–1.79	45/16	1.77	1.54–4.82
	Sulpiride	7,600	126/91	1.32*	1.01–1.72	112/65	1.36	1.21–2.22
**Drugs for peptic ulcer**	Cimetidine	2,886	53/25	1.99*	1.24–3.20	48/18	1.72	1.46–4.30
	Scopolamine butylbromide	97,755	909/523	1.72*	1.55–1.92	703/391	1.13	1.57–2.02
**Anticholinergic bronchodilator**	Tiotropium bromide	5,179	234/135	1.75*	1.42–2.16	213/94	1.27	1.80–2.92
**Antihistaminic**	Chlorpheniramine	3,934	6/7	0.87	0.29–2.60		3.72	0.342–4.74
	Diphenhydramine	13,073	0/2	NA	NA	4/5	NA	NA
**Antiarrhythmics**	Cibenzoline	2,038	81/27	2.97*	1.92–4.59	73/21	1.62	2.12–5.59
**Cardiac stimulants excl. cardiac glycosides**	Amezinium metilsulfate	1,665	38/20	1.89*	1.10–3.26	34/13	1.89	1.38–4.94
	Droxidopa	650	15/17	0.90	0.45–1.79	13/11	2.23	0.537–2.68
	Midodrine	1,346	24/20	1.21	0.67–2.20	23/12	2.01	0.964–3.89
**Opioids**	Morphine	20,378	599/449	1.29*	1.14–1.45	564/370	1.14	1.29–1.68
	Codeine phosphate	11,223	234/223	1.04	0.86–1.25	192/137	1.25	1.11–1.72
	Oxycodone	6,370	334/289	1.20*	1.03–1.41	333/176	1.2	1.64–2.37

NA: Not applicable. *Statistical significant at an alpha level of 0.05.

Index → Marker (causal): patients initiated marker drug therapy after initiating index drug treatment.

Marker → Index (noncausal): patients initiated index drug therapy after initiating marker drug treatment.

Index drug: Medicine associated with a risk of LUTS. Marker drug: Medication for treating LUTS.

Number of participants who initiated a medicine associated with LUTS between 2005 and 2013.

Sequences of the incident index and marker drugs within 3 or 12 months.

Adjusted for the null-effect SR.

## Discussion

The number of prescription drugs, but not age, was significantly different between users (12.13 ± 6.70) and nonusers (5.67 ± 5.24) of medicines that can cause urinary symptoms.

Polypharmacy, inappropriate prescribing, and adverse drug events are highly prevalent in elderly patients [23]. The characteristics of patients with LUTS included old age (average age, 70.52 ± 14.60) and male sex. Voiding symptoms was noted in 57.9% of women, presumably because of declines in muscle strength, such as pelvic floor muscle strength, with age. In men, the prevalence of high outlet obstruction (91.5%) predominated as a result of prostate enlargement with age.

The typical elderly patient with LUTS typically receives medication for other comorbidities. Elderly patients taking five to eight drugs were reported to be at greater risk of adverse drug reaction related hospitalization than those taking zero to four drugs [23]. However, a meaningful correlation was not found between age and the number of the prescription drugs used by patients with LUTS (data not shown) in this study. This result may be responsible for the findings that patients with LUTS were old (average age, 70.52 ± 14.60) and presented with several comorbid conditions requiring medication (average number of prescription drugs, 6.16 ± 5.38).

In total, 7.7% of patients were prescribed medications potentially contributing to urinary symptoms. Donepezil (14.1%), antiparkinson drugs (13.7%), antidepressants (13.8%), and antipsychotics (13.3%) were frequently prescribed, reflecting the rate of complications in older people. Patients who used medicines that can cause storage symptoms, such as donepezil, were older than nonusers. In contrast, patients who used medicines that can cause voiding symptoms, such as antiparkinson drugs (13.7%), antidepressants (13.8%), and antipsychotics, were younger than their nonusing counterparts. The prevalence of incontinence in the elderly with dementia is higher than that of persons without dementia. Multiple comorbid diseases, such as depression, Parkinson’s disease, psychotic disorders, and medications are possible risk factors for voiding symptoms.

In this study, we demonstrated that the side effects of donepezil, cyclophosphamide, amantadine, levodopa/benserazide, paroxetine, fluvoxamine,milnacipran, diazepam, risperidone,levomepromazine,sulpiride, cimetidine, scopplamine butylbromide, tiotropium bromide, and opioids were associated with LUTS. Risperidone therapy has been associated with storage symptoms (28% in some cases) [24]. Risperidone primarily acts as an antagonist of serotonin type 2A (5HT2a) and D2-dopamine receptors, and it has a strong blockade effect on α-1 and α-2 adrenergic receptors. It may be that the antagonizing effect of risperidone on the α-1 receptors of the internal bladder sphincter causes urinary retention [25]. Levomepromazine also may affect the α-1 receptors of the internal bladder sphincter.

Sulpiride, a D2-selective dopaminergic receptor antagonist, increased bladder capacity in rats [26]. Dopaminergic systems have also been implicated in the control of the micturition reflex. D1-like dopaminergic receptors mediate forebrain-inhibitory effects on the micturition reflex, whereas D2-like dopaminergic receptors appear to be involved in excitatory regulation of the micturition reflex at the level of the brainstem. The prevalence of LUTS in patients with Parkinson disease (PD) is reported to be 27%–39% [27]. Levodopa is well established as the most effective drug for the symptomatic treatment of idiopathic or Lewy body PD. Amantadine is known to increase dopamine release, inhibit dopamine reuptake, and stimulate dopamine receptors, and it may possibly exert central anticholinergic effects [27].

Anticholinergic agents such as biperiden and trihexyphenidyl did not display meaningful peripheral antimuscarinic side effects. In contrast, the dopamine-related drugs levodopa and amantadine exerted side effects, suggesting that LUTS caused by such drugs is dependent on dopaminergic activity.

Milnacipran, a dual-action antidepressant that acts as a serotonin and norepinephrine reuptake inhibitor (SNRI), and paroxetine, a selective serotonin reuptake inhibitor (SSRI), have been linked to voiding symptoms. Animal studies suggested that incontinence secondary to serotonergic antidepressants could be mediated by 5HT4 receptors found on the bladder [28,29]. These drugs may affect serotonin uptake by 5HT receptors found on the bladder. Recently, experiments in cats illustrated that SNRIs suppress parasympathetic activity and increase sympathetic and somatic neural activity in the lower urinary tract [30]. The inhibition of serotonin and norepinephrine reuptake during bladder storage is believed to increase pudendal nerve output, resulting in increased tone of the rhabdosphincter and subsequently improved urethral closure. The SNRI duloxetine can significantly improve the quality of life of patients with stress urinary incontinence. In this study, voiding symptoms was caused by milnacipran, suggesting that milnacipran-associated voiding symptoms may have beneficial effects on urinary incontinence.

The most common medication class potentially contributing to LUTS is benzodiazepines (17.4%) [31]. The mechanism by which other medicines cause incontinence is the activation of *N*-methyl-*D*-aspartate receptors in pontine micturition center (PMC) that are involved in the facilitation of voiding [32].

Men with LUTS or BPH who are treated with inhaled anticholinergic agents may develop acute urinary retention, but this cannot be quantified on the basis of the limited information available. Inhaled anticholinergic agents should be used when indicated in men with LUTS or BPH but close monitoring and patient education should be implemented [33]. Inhaled anticholinergic medication use in older men with chronic obstructive pulmonary disorder is associated with an increased risk of acute urinary retention [34].

The prevalence of opioid-induced dysuria in patients with advanced cancer-associated pain was 14.9% [35]. Urinary retention induced by systematically injected morphine was considered to result from the inhibition of bladder function mediated via μ-opioid receptors of the micturition centers in the supraspinal and spinal regions [36].

Dementia and storage symptoms are common, and often coexisting, problems in older people. Anticholinergic drugs are used to treat urinary instability [37-39]. In this study, storage symptoms is expected to occur in patients treated with donepezil. The potential for worsened urinary continence is an important consideration when starting cholinesterase treatment in Alzheimer disease (AD).

Cyclophosphamide is metabolized to several moieties, including acrolein, which collects in the urinary bladder, leading to urothelial damage [40]. Cyclophosphamide causes cellular damage in bladder tissue that triggers an inflammatory response. Therefore, cyclophosphamide is commonly used in noninvasive rodent models of acute bladder pain [41].

The current study has highlighted the potential for the initiation of commonly used medicines to be associated with the subsequent initiation of drug treatment for LUTS and has provided an estimate of the risk of LUTS associated with these medicines. Prescribers should be aware of LUTS that occurs shortly after the initiation of new medicines, and the potential for an adverse event should be considered. For this reason, before the treatment for LUTS is started, the possible side effects of medications currently used for the patient should be reviewed and the planned treatment for LUTS should be adjusted if necessary.

## Conclusions

We found that medicines that can cause urinary symptom were prescribed to patients taking many drugs and were associated with an increased risk of LUTS. The findings illustrated that polypharmacy and adverse drug events associated with LUTS-causing drugs were highly prevalent in patients with LUTS. In addition, our study provides a basis for future investigations of whether milnacipran-associated voiding symptoms may be useful for the treatment of storage symptoms.

## Additional file

Additional file 1:
**Medication for LUTS.**
