# Supplementary material for: Prescription rate of medications potentially contributing to lower urinary tract symptoms and detection of adverse reactions by prescription sequence symmetry analysis
Source: J Pharm Health Care Sci. 2015 Feb 15;1:7. doi: 10.1186/s40780-014-0004-1 (PMC4728807; doi:10.1186/s40780-014-0004-1)
Supplement: Additional file 1: — Medication for LUTS. [file 40780_2014_4_MOESM1_ESM.doc]

# Additional file

### Additional file 1 – Medication for LUTS

| **Disease** | **Medication** |
| --- | --- |
| Urge incontinence | Oxybutynin |
|  | Propiverine |
| Overactive bladder | Flavoxate |
|  | Tolterodine |
|  | Solifenacin |
|  | Imidafenacin |
| Abdominal pressure‐induced incontinence | Clenbuterol |
| Overflow incontinence | Bethanechol |
|  | Distigmine |
|  | Neostigmine |
| Dysuria associated with benign prostatic hyperplasia (BPH) | Paraprost |
|  | Cernitin pollen extract |
|  | Eviprostat |
|  | Tamsulosin |
|  | Naftpidil |
|  | Silodosin |
|  | Prazosin |
|  | Urapidil |
|  | Terazosin |
| Enuresis | Imipramine |
|  | Clomipramine |
| Nocturnal enuresis | Amitriptyline |
